# Hybrid Effect of Twisted Steel and Polyethylene Fibers on the Tensile Performance of Ultra-High-Performance Cementitious Composites

**DOI:** 10.3390/polym10080879

**Published:** 2018-08-06

**Authors:** Min-Jae Kim, Soonho Kim, Doo-Yeol Yoo

**Affiliations:** Department of Architectural Engineering, Hanyang University, 222 Wangsimni-ro, Seongdong-gu, Seoul 04763, Korea; mandufa345@hanyang.ac.kr (M.-J.K.); tnsgh0905@hanyang.ac.kr (S.K.)

**Keywords:** fiber aspect ratio, hybrid reinforcement, mechanical property, polyethylene fiber, twisted steel fiber, ultra-high-performance cementitious composite

## Abstract

The hybrid effect of twisted steel (T) fibers with an aspect ratio of 100 and polyethylene (PE) fibers with four different aspect ratios of 400, 600, 900, and 1200 on the mechanical performance of ultra-high-performance cementitious composite (UHPCC) was investigated. This involved a total of 17 different sample types at an identical fiber volume fraction of 2% being made and subjected to compressive and tensile loads. Samples were made by replacing 0.5%, 1.0%, 1.5%, and 2.0% of T fibers with four different types of PE fibers. In addition, the pullout behaviors of fibers at cracked sections and the cracking behaviors of specimens were evaluated in order to determine the effect of the pullout mechanism of each fiber on the overall tensile performance. Test results indicate that the compressive strength decreased in proportion to the amount of PE fibers, regardless of their aspect ratio. The fiber hybridization had a great synergetic effect, successfully improving the tensile strength and strain capacity of UHPCCs; this effect was dependent on the aspect ratio of the PE fibers. Finally, the cracking behaviors were determined to be more related to the fiber type and pullout mechanisms than the tensile strength or strain capacity of UHPCCs.

## 1. Introduction

The two most widely used strain-hardening high-performance cementitious composites were the ultra-high-performance cementitious composites (UHPCCs) and engineered cementitious composites (ECCs). Both of these material types show excellent mechanical performance. UHPCCs demonstrate specialized performance, including very high compressive strength at over 150 MPa, designed tensile strengths of 8 MPa, and strain capacity of 0.5% [1,2,3,4], which is relatively much smaller than that of ECCs. Alternatively, ECCs generally exhibit compressive strengths of 30–80 MPa, tensile strengths of 5–8 MPa, and very large strain capacities approaching 5% [5]. The former material type normally incorporates various types of steel fibers to improve its mechanical properties, especially in terms of tensile performance. Thus, a significant amount of research has already been conducted in an attempt to enhance the pullout resistance of steel fibers and the tensile or flexural performance of UHPCCs. Since the mid-2000s, the effect of geometric deformation of steel fibers has been investigated as an efficient way to improve the mechanical properties of UHPCCs [6,7,8,9]. The most broadly used deformed steel fibers are classified into two types: Twisted steel (T) and hooked-end steel fibers. The T fiber, which was first introduced by Namman in 1998 [10], has shown the best efficiency in enhancing the tensile performance of high-performance cement composites [10,11]. Another group of studies [3,8] also reported that the geometric deformation of steel fibers tends to damage the cement matrices of UHPCCs when they are straightened under pullout loads. According to Yoo et al. [3], the post-peak flexural stress of UHPCCs containing deformed steel fibers decreased more radically compared to other specimens with the same amount and length of straight steel fibers; this difference in performance is due to matrix damage caused by T fibers. Wille et al. [8] also found that the geometric deformation produces severe stress concentrations, causing the matrix and its resistance to pullout to undergo severe deterioration. Thus, work still needs to be done to address the detrimental effect of deformed steel fibers in UHPCCs in order to maximize their reinforcing efficiency. For ECCs, however, polymeric fibers (e.g., polypropylene (PP), polyethylene (PE), and polyvinyl alcohol (PVA) fibers) are generally incorporated to enhance the ductility of cement composites [5]. Typical PE–ECC or PVA–ECC that has been properly treated (e.g., subjected to a surface coating treatment using plasma or oiling agents) normally show 4–5% strain capacity with normal strength levels. Thus, the strength values of ECCs are smaller than those of UHPCCs. Therefore, it is obvious that there are still some limitations for both UHPCCs and ECCs, such as the low ductility and low strength, respectively.

In this study, in order to overcome the limitations of UHPCCs and ECCs, a fiber-hybridizing technique was adopted to obtain high-strength and high-ductility properties in cementitious composites. To achieve this, the synergetic effects of different types of fibers on the mechanical properties need to be analyzed. The steel and polymeric fibers have different geometries and physical properties. In addition, cement composites consist of various substances with multi-phases and different sizes, such as C–S–H gel, silica sand, fillers, etc., as was reported by Yao et al. [12]. Therefore, the hybrid effects of two different fibers, i.e., steel and polymeric fibers, are worth investigating. To investigate the synergetic effect of hybrid fibers, T fibers and PE fibers with four different aspect ratios were hybridized at the same mixture proportion of UHPCCs, while also maintaining an overall fiber volume fraction of 2%. T fibers were adopted in this study based on the results of a previous study [8]; UHPCCs with T fibers exhibited better tensile performance than composites with straight or hooked-end steel fibers. However, further enhancement of the tensile performance of UHPCCs with T fibers has been limited due to the formation of splitting cracks in the cement matrix surrounding the T fibers; these are caused by the excellent bond strength and untwisting moment of T fibers. For this reason, our study focused on mitigating the premature deterioration of the matrix caused by the pulling-out process of T fibers. This was done by replacing a portion of the T fibers with PE fibers, which have superb tensile strength, to prevent rupture before complete pullout. Subsequently, the effect of the PE fiber aspect ratio on the tensile performance of UHPCCs with hybrid reinforcements of T and PE fibers was investigated. 17 different specimens incorporating a total of 2 vol % fibers were considered, and the T fibers were replaced with 0.5, 1, 1.5, and 2 vol % of four types of PE fibers. Also, as control specimens, five non-hybrid specimens with only T or PE fibers with different aspect ratios were adopted. During the tensile tests, micrography was conducted at the localized crack surfaces to rationally analyze the tensile test results based on the pullout mechanism of each fiber.

## 2. Experimental Program

### 2.1. Mix Proportions of UHPCCs

The mixture proportion of UHPCC adopted in this study is described in Table 1. Type 1 Portland cement and silica fume were used as cementitious materials; these have the chemical compositions and physical properties summarized in Table 2.

The specific surface areas of the cement and silica fume are 3413 and 200,000 cm^2^/g, respectively, and their densities are 3.15 and 2.10 g/cm^3^, respectively. Silica sand and silica flour with granular sizes of 0.2–0.3 mm and 10 μm were adopted as an aggregate and a filler, respectively, based on the packing density theory and preliminary rheological and mechanical test results [13]. It should be noted that a coarse aggregate was not added into the mixture. Several previous research works have found that including coarse aggregates in UHPCCs leads to positive effects in terms of the cost effectiveness, high fluidity, less mixing time without any deteriorations in the compressive strength, and shrinkage reduction [14,15]. However, in accordance with previous literature [1,16], the fiber-matrix bonding properties and flexural performance of UHPCCs are deteriorated due to the reduction of matrix shrinkage caused by including coarse aggregates. In addition, it is well-known that eliminating coarse aggregates makes the cement composites more compact and homogeneous, resulting in better mechanical properties [17]. These are the main reasons that coarse aggregate was eliminated from the mixture proportions in this study. A very low water–to–binder (W/B) ratio of 0.2 was applied to the mixture, while a variety of fine granular constituents with high specific surface areas were incorporated to achieve better mechanical properties in the UHPCCs. A polycarboxylate superplasticizer with a density of 1.01 g/cm^3^ was used to obtain sufficient fluidity in the low W/B ratio, and its fluidity was measured according to ASTM C1437 [18]. The fluidity significantly decreased upon the inclusion of PE fibers in proportion to the volume fraction; the content of the superplasticizer was adjusted to obtain proper fluidity, as summarized in Table 3.

Physical properties of the T fibers and four types of PE fibers were summarized in Table 4. The T fiber with a length of 30 mm and PE fibers with four different lengths of 12, 18, 27, and 36 mm, and the four types of PE fibers described above are referred to as SPE, MPE, LPE, and LLPE fibers, respectively. The T or the SPE, MPE, LPE, and LLPE fibers have aspect ratios of 100 or 400, 600, 900, and 1200, respectively. Tensile strengths of the T and the PE fibers are 2788 and 3030 MPa, and the measured elastic moduli were 200 and 88 GPa, respectively. The T fiber had a triangular cross-sectional shape and was twisted three times along the longitudinal direction to obtain additional mechanical anchorage effect during its pullout [10]. Alternatively, the PE fibers were straight and had a circular cross-sectional shape.

A total of 17 types of specimens incorporating the same amount (2% by volume) of non-hybrid or hybrid T and the four types of PE fibers were fabricated and tested: 5 non-hybrid specimens including 2 vol % T, SPE, MPE, LPE, and LLPE fibers and 12 hybrid specimens incorporating combinations of the T and the PE fibers (i.e., T0.5-(S, M, L or LL)PE1.5, T1.0-(S, M, L, or LL)PE1.0, and T0.5-(S, M, L, or LL)PE1.5), were also fabricated to investigate the effect of fiber hybridization. For convenience, the specimens can be divided into 5 series, i.e., T2.0, T0.5-PE1.5, T1.0-PE1.0, T1.5-PE0.5, and PE2.0 series, and more specifically, the four series which incorporating a portion of PE fibers include four specimens (e.g., the T0.5-PE1.5 series includes T0.5-SPE1.5, T0.5-MPE1.5, T0.5-LPE1.5, and T0.5-LLPE1.5 specimens). The letters “T” and “(S, M, L, or LL)PE” denote T and PE fibers, respectively, and the subsequent numbers denote the fiber volume fraction. Accordingly, a specimen reinforced with 2 vol % T fibers is referred to as T2.0 and a specimen with 0.5% T fibers and 1.5 vol % SPE fibers is referred to as T0.5-SPE1.5.

### 2.2. Mixing Process and Specimen Fabrication

To obtain good-quality UHPCCs, special mixing procedures were applied. First, the dry materials (i.e., Portland cement, silica fume, silica sand, and silica flour) were slowly premixed for 5 min. Then, water and the superplasticizer were added and the mixture was mixed for an additional 10 min until it obtained sufficient fluidity. Finally, previously weighed fibers were gradually poured into the mixture and an additional 5 min of mixing was conducted; the PE fibers were premixed with the dry materials prior to the inclusion of the water and superplasticizer to obtain better dispersion. After completing the whole mixing process, the mixture was placed into molds and compacted for a minute with a vibrator, covered with a plastic sheet to prevent drastic shrinkage caused by evaporation, and cured for 24 h at room condition with an average temperature of 20 °C and a relative humidity of 60%. Then, the specimens were demolded and steam cured at 90 °C for 72 h to promote the hydration reaction and strength development [10]. After the steam curing, the specimens were placed in the room condition, and all the mechanical tests were performed at the age of 7 days after casting.

### 2.3. Experimental Setups for Compressive and Tensile Tests

The compressive strength test was performed by using cylinders with a dimension of Φ100 × 200 mm^2^, as recommended by ASTM C39 [19]. To minimize the eccentric effect on the compressive strength, the casting surface of the cylindrical specimens was ground flat using a diamond blade. A uniaxial load was monotonically applied using a universal testing machine (UTM) with a maximum load capacity of 300 tons. To ensure the accuracy of the test, three samples for each variable were tested and averaged.

**Figure 1:**
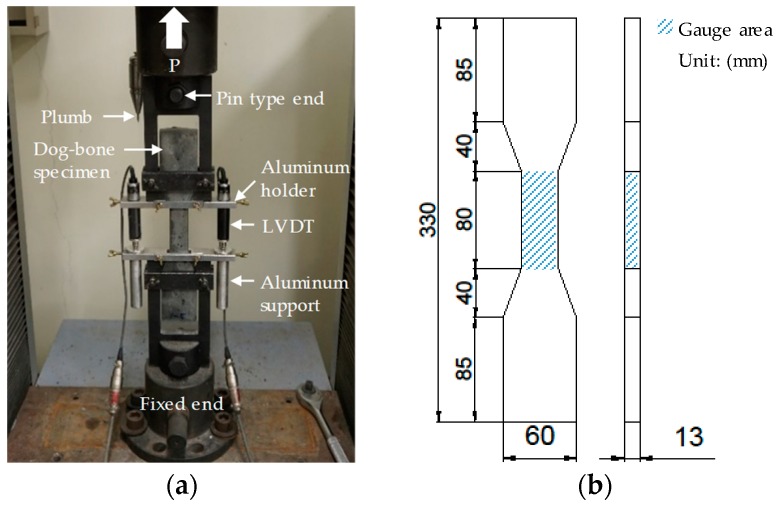
Tensile test (**a**) setup (**b**) geometrical details of specimen.

Uniaxial tensile tests were performed in accordance with JSCE recommendations [20]. The test setup and geometry of a tensile specimen are illustrated in Figure 1. A UTM with a loading capacity of 300 kN which was produced by Shimadzu Corp. (AG-300KMX, Kyoto, Japan) was utilized for the tensile test. A load cell and two linear variable displacement transducers (LVDTs) were installed, as shown in Figure 1a, to measure the tensile load and displacement, respectively. The measured load and displacement were transferred to the tensile stress and strain of the gauge area with a cross sectional area of 30 × 13 mm^2^ and a height of 80 mm shown in the Figure 1b. The uniaxial load was applied based on a displacement control with a loading rate of 0.4 mm/min. Five specimens were tested for each variable and the results were averaged to obtain reliable and representative values.

## 3. Test Results and Analysis

### 3.1. Compressive Strength

**Figure 2:**
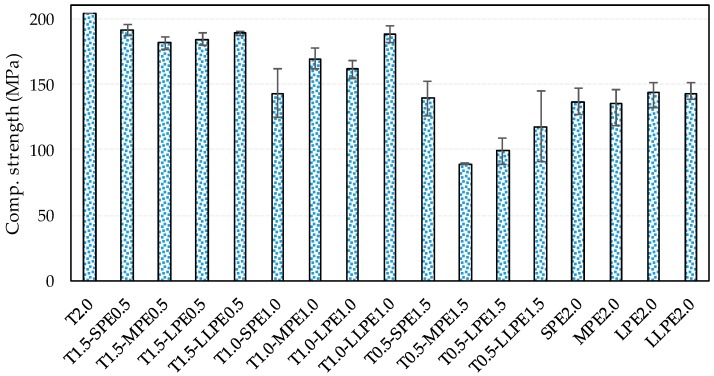
Compressive strength test results.

The compressive strength data of all tested specimens are summarized in Figure 2. The effect of fiber types on the compressive strength are generally less than the tensile properties of UHPCCs [6,21]. Based on the test results of this study, however, it was found that the PE fibers noticeably deteriorate the mixture fluidity and compressive strength. The highest compressive strength of the T2.0 specimen was found to be 204.4 MPa, and the compressive strength decreased with an inclusion of PE fibers. The decrease also increased in proportion to the content of PE fibers. The compressive strengths of the T2.0, T1.5-PE0.5, T1.0-PE1.0, T0.5-PE1.5, and PE2.0 specimen series were measured as 204.4, 186.6 (4.6), 165.6 (18.6), 111.3 (22.2) and 139.5 (7.8) MPa on average, respectively. The results of the four series except for the T2.0 are average values derived from four lengths of PE fibers with different aspect ratios. The numbers in parentheses indicate the standard deviations resulted from the different length of the PE fibers. It is noticed that the standard deviations increased in proportion to PE fiber hybrid ratio, whereas that of the PE2.0 series was relatively minor. This demonstrates that the compressive strength varied more irregularly and largely in the hybrid systems according to the increase in length and content of PE fibers. This can be verified in Figure 2, where it is shown that the maximum compressive strength differences in the T1.0-PE1.0 and the T0.5-PE1.5 series were measured to be 31.3% and 56.5%, while those for the T1.5-PE0.5 and the PE2.0 series were only 5.7% and 6.4%, respectively. According to a previous report [22], T fibers showed positive effectiveness in enhancing compressive strength of UHPCCs. In this study, however, the T0.5-PE1.5 series showed the average compressive strength of 111.3 MPa, which was 32.5 MPa lower than that of the PE2.0 series. Only the hybrid specimen with short PE fibers (T0.5-SPE1.5) showed the compressive strength of 139.3 MPa, which was similar to that of the SPE2.0 specimen. Thus, it can be noted that hybridizing the T and PE fibers could further deteriorate the dispersion and orientation of PE fibers due to their different physical and chemical properties, and thus the compressive strength decreased [23,24] as compared to the single PE2.0 series.

### 3.2. Hybrid Effect of T and PE Fibers on the Tensile Properties

#### 3.2.1. Tensile Stress–Strain Behavior: Post-Cracking Stiffness and Post-Peak Ductility

**Figure 3:**
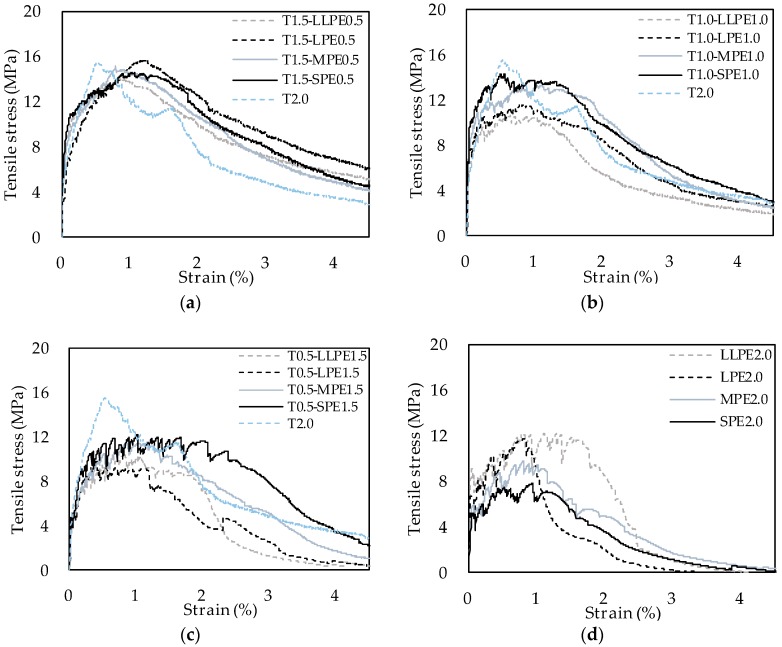
Tensile stress versus strain curves of (**a**) T0.5-PE1.5; (**b**) T1.0-PE1.0; (**c**) T1.5-PE0.5; and (**d**) PE2.0 series of specimens.

Figure 3 shows the tensile stress versus strain curves of all of the specimens tested. Post-cracking stiffness, which is a slope between the first cracking point and the peak point, of the T2.0 specimen was the highest in this study. As Li and Leung [25] reported, stronger interfacial bond between fiber and matrix leads to shorter strain hardening behavior. The T fiber has a strong chemical bond and mechanical bond with the matrix which derive a strong interface [3,8]. Accordingly, the T2.0 specimen showed the highest post-cracking stiffness, as shown in Figure 3a–d. Post-cracking stiffness of the T2.0 specimen, however, significantly decreased with an inclusion of PE fibers. When 0.5% (by volume) of T fibers were replaced with PE fibers (Figure 3a), the post-cracking stiffness similarly decreased regardless of length of PE fibers, because the stress-strain curves did not considerably differ from each other. However, differences derived from length of PE fibers became clear as their volume contents increased to 1.0% and 1.5% as shown in Figure 3b,c. It is noticed that the tensile strengths generally descended as the length of PE fiber increased from 12 (SPE) to 36 (LLPE) mm. Thus, the post cracking stiffness decreased larger with an inclusion of longer PE fibers in steel–PE hybrid systems. In contrast, for the PE2.0 series, the tensile performance was enhanced overall with an increase in length of PE fibers. Differences in post-cracking stiffness derived from length of PE fibers, however, were comparatively lower than those measured in hybrid systems as shown in Figure 3d.

Figure 3a shows that post-peak ductility of the T2.0 specimen was the poorest showing drastic decrease after the peak point. As mentioned previously, the T fibers damage the matrix by untwisting torque derived from its geometrical deformation and mechanical bond. This damaged the matrix with causing it to lose its pullout resistance and tensile stress to drastically decrease as reported in previous study [3,8]. With an inclusion of PE fibers, on the other hand, decrease in tensile stress became more gradual after the peak point (Figure 3a). There was no discernable difference in the post-peak ductility according to the length of PE fibers. In addition, the stress-strain curves became flatter and fluctuated more as the content of PE fibers increased to 1.0% and 1.5%, rather than decreasing drastically. These trends became more significant when 1.5% of SPE and MPE fibers were used than the longer fibers, resulting in more obvious strain hardening behavior as shown in Figure 3b,c. Thus, it can be noted that the shorter PE fibers are effective in enhancing strain hardening behavior of steel-PE hybrid systems. Meanwhile, the fluctuation of stress-strain curves became clearer when the longer PE fibers were used in the PE2.0 series, which is a single system, as shown in Figure 3d. In addition, the LLPE fiber specimens resulted in the greatest fluctuation and strain capacity.

#### 3.2.2. Tensile Strength and Strain Capacity

**Figure 4:**
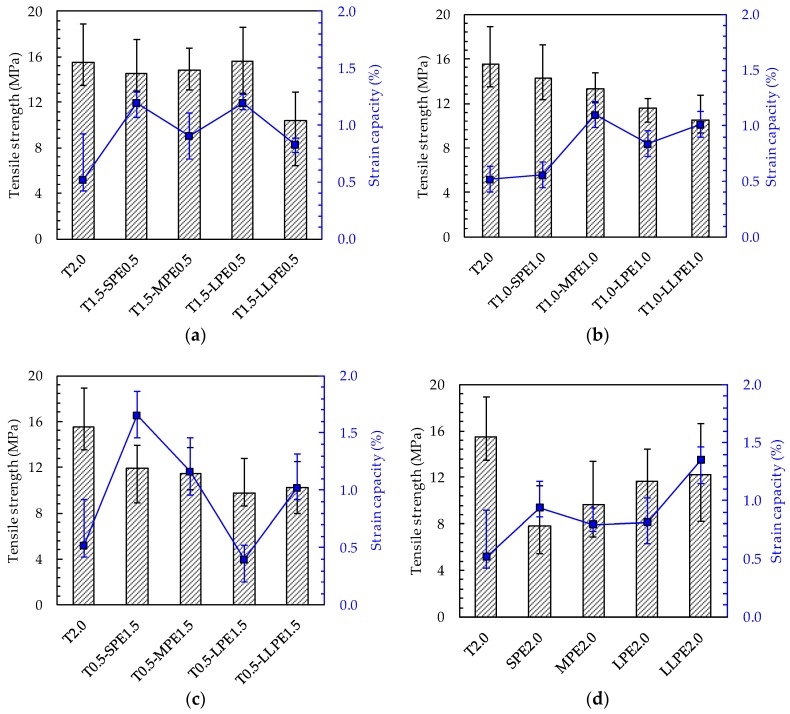
Tensile strength and strain capacity of (**a**) T0.5-PE1.5; (**b**) T1.0-PE1.0; (**c**) T1.5-PE0.5; and (**d**) PE2.0 series.

**Figure 5:**
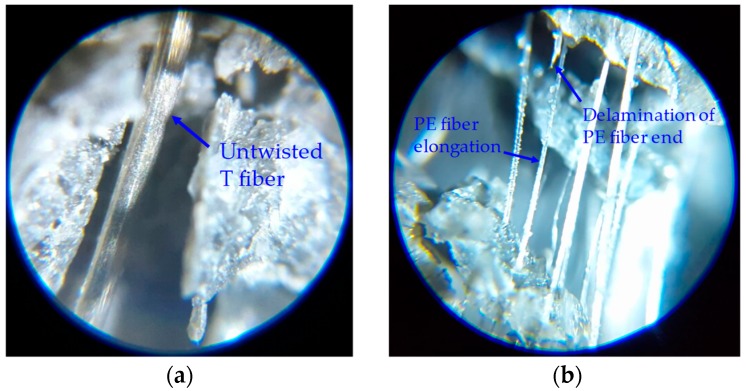
Microscopic images of UHPCC: (**a**) A untwisted T fiber and (**b**) elongation of LLPE fibers.

Figure 4 shows the tensile strength and strain capacity values of all the specimens, respectively. The strain capacity is a strain value measured at the peak point, which is a strain value of the gauge area corresponding to the tensile strength. The T2.0 specimen provided a very high tensile strength of 15.5 MPa and a relatively low strain capacity of about 0.52%. Such a high tensile strength was mainly caused by the strong pullout resistance of the T fibers. The T fibers are designed to provide the torsion along their length as they are pulled out of the matrix, and this untwisting action requires the matrix to provide more resistance, leading to the strain–hardening behavior [11]. However, it has also been reported that the tensile or flexural stress of UHPCCs containing T fibers decreased more rapidly after the peak point compared to those containing straight steel fibers [7,22]. Previous studies [7,22] found that the T fiber splits the surrounding cement matrix when it is pulled out due to its untwisting moment and it is not fully untwisted even after complete pullout from the UHPCC matrix. Figure 5 shows microscopic images at a localized crack surface; the image shows T and PE fibers that were partially pulled out from the matrix. From the Figure 5a, it is shown that the T fiber was not fully untwisted and the matrix was split before the T fiber was fully pulled out. Therefore, pullout resistance of the matrix was weakened before reaching its maximum capacity causing the T fiber to be pulled out easily and the post-peak tensile stress of UHPCCs to decrease steeply upon crack localization.

When 0.5% of the T fibers were replaced with PE fibers (i.e., the T1.5-PE0.5 series), no significant decreases were found in the tensile strength, but the strain capacity largely improved, ranging from 0.83% to 1.19%. This is approximately two times that of the T2.0 specimen. However, the T1.5-LLPE0.5 specimen showed poor tensile performance, which might be derived from the poor fiber dispersion. It is worth noting that, for the T1.5-PE0.5 series (Figure 4a), the tensile strength continuously increased from 14.5 to 15.6 MPa as the PE fiber length increased from 13 (SPE) to 27 mm (LPE). Alternatively, when the 36-mm-long LLPE fibers were included, the tensile strength and strain capacity largely decreased to 10.4 MPa and 0.83%, respectively. For the T1.0-PE1.0 series (Figure 4b), the tensile strength decreased from 14.3 MPa to 10.5 MPa as the length of the PE fibers increased from 13 mm (SPE) to 36 mm (LLPE), while the strain capacity varied irregularly between 0.56% and 1.10%. The T1.0-SPE1.0 specimen showed the highest tensile strength but the lowest strain capacity, whereas the T1.0-LPE1.0 and T1.0-LLPE1.0 specimens showed lower tensile strengths of 11.6 and 10.5 MPa but higher strain capacities of 0.84% and 1.01%, respectively. For the T0.5-PE1.5 series (Figure 4c), both the tensile strength and strain capacity decreased with increasing PE fiber length, ranging from 9.8 to 11.9 MPa and from 0.40% to 1.66%, respectively. The highest strain capacity of 1.66%, about 3 times greater than that of the T2.0 specimen, was found in the T0.5-SPE1.5 specimen, but its tensile strength was slightly lower than that of the T2.0.

Based on the test results, three important findings are listed as follows. First, the tensile strength of the UHPCC was proportional to the volume content of T fibers. The average tensile strengths of each series were measured to be 10.8, 12.4, 13.8, and 15.5 MPa for the specimens containing 0.5, 1.0, 1.5, and 2.0 vol % T fibers, respectively. This indicates that the T fibers are more efficient in increasing the tensile strength of UHPCCs than the PE fibers. Second, as the volume fraction of PE fibers increases, the tensile performance of UHPCCs was generally deteriorated with an increase in the aspect ratio of the PE fibers. In the T1.5-PE0.5 series, no clear difference in the tensile performances derived from the aspect ratio of PE fibers was observed. However, in the T1.0-PE1.0 and T1.5-PE0.5 series, the SPE and the MPE fibers were more effective in improving the tensile performance of UHPCCs than the LPE and the LLPE fibers. Lastly, the tensile strength and strain capacity of the PE2.0 series were enhanced effectively by increasing the aspect ratio of PE fibers, as shown in Figure 4d. The tensile strengths of the SPE2.0, MPE2.0, LPE2.0, and LLPE2.0 specimens proportionally increased from 7.8 to 12.2 MPa, and the strain capacity also generally increased from 0.79% to 1.35% as the length of the PE fiber increased up to 36 mm. This is attributed to the fact that fibers with a higher aspect ratio more effectively enhance the post-cracking tensile strength of UHPCC because it is proportional to the fiber-reinforcing index: *v_f_ × l_f_/d_f_* [1,3,26], where *v_f_* is the fiber volume fraction, *l_f_* is the fiber length, and *d_f_* is the fiber diameter. It was found that the LLPE fibers with the highest aspect ratio of 1200 did not rupture after the tensile test, although some of the PE filaments of the fibers were partially delaminated, as shown in Figure 5b. Thus, the tensile strength could be improved by increasing the aspect ratio of the PE fibers. This is inconsistent with the trend observed for the hybridized series of specimens discussed above, and this difference is caused by the different pullout mechanisms of T and PE fibers and poorer dispersion of PE fibers with higher aspect ratios due to the disturbance of T fibers existed. The degree of disturbance caused by the T fibers on the dispersion of PE fibers became smaller as the aspect ratio of the PE fibers decreased, so that shorter PE fibers with lower aspect ratios might be more evenly dispersed in the matrix alongside the T fibers. This was responsible for the improved mechanical performance of UHPCCs, similar to the results of previous studies [3,27].

The T fibers tend to split the matrix during their untwisting process, and they can also easily mash the fine particles stacked up at the interface with matrix due to their relatively high surface hardness. Alternatively, the PE fibers does not damage the matrix due to their very low surface hardness. Therefore, T fibers were more easily slipped once the splitting cracks were formed in the surrounding matrix, while it is not easy for the PE fibers to be pulled out because of strong and continuous frictional pullout resistance given by the particles at the interface. In addition, the T fibers have an elastic modulus of 200 GPa which is 2.3 times that of the PE fibers (88 GPa). So, when a tensile load was applied to the composite, T fibers tend to slip rather than elongate after full debonding from the matrix, whereas the PE fibers tend to stretch more due to their lower elastic modulus and the strong and continuous frictional resistance from the matrix particles. This led to greater strain capacity of UHPCCs with PE fibers than that with only the T fibers at an identical tensile load. In addition, the splitting cracks in the matrix surrounding the T fibers were more effectively resisted by PE fibers when shorter PE fibers were used, caused by their higher numbers as compared to those of the longer fibers. Thus, the shorter PE fibers were more effective in preventing premature deterioration of the matrix and enhancing the synergy effect with the T fibers.

#### 3.2.3. Energy Absorption Capacity per Unit Volume

**Figure 6:**
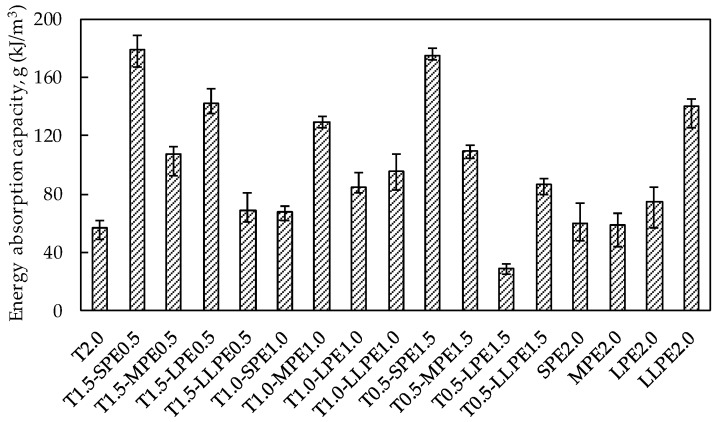
Energy absorption capacity analysis up to the peak point of UHPCCs.

In this study, the energy absorption capacity per unit volume up to the peak point (denoted as the *g*-value, in a unit of kJ/m^3^) was calculated. Wille et al. [2] recommended the *g*-value to be used as a performance evaluation index. They defined 50 kJ/m^3^ as a standard value for fiber-reinforced concrete (FRC) with the highest performance (i.e., level 4), while the lowest level 0 indicates normal concrete without fibers. The *g*-values for all the specimens were illustrated in Figure 6. Every specimen dissipated more than 50 kJ/m^3^, except only for the T0.5-LPE1.5 specimen, which showed abnormally low tensile performance.

The average *g*-value of the T2.0 specimen was found to be 56.6 kJ/m^3^, which was lower than the energy absorption capacities obtained by the other hybrid and PE specimens. As shown in Figure 3, the tensile stress of the T2.0 specimen rapidly increased up to its peak strength of 15.5 MPa, due to the strong chemical bonding with cement matrix and mechanical anchorage effects generated by three rips of the T fibers. Alternatively, all of the PE2.0 series (i.e., SPE2.0, MPE2.0, LPE2.0, and LLPE2.0) showed a gradual increase in tensile stress after the first cracking point up to the peak point. Even though the tensile strength was much lower than that of the T2.0 specimen, all of the PE2.0 series dissipated greater amount of energies ranging from 59.8 to 139.9 kJ/m^3^. This was because the strain capacities were very high, ranging from 0.79% to 1.35%. It can be noticed that the longer PE fibers showed greater efficiency in enhancing the energy absorption capacity under tension when they were singly used.

The tensile performance of the SPE2.0 and MPE2.0 specimens were largely improved as a portion of the PE fibers were replaced with the T fibers. For instance, the tensile strength of the SPE2.0 and MPE2.0 specimens were 7.8 and 9.7 MPa, respectively, and the strain capacities were 0.94% and 0.79%, respectively. However, the T0.5-SPE1.5 and T0.5-MPE1.5 specimens provided tensile strengths of 11.9 and 11.5 MPa and strain capacities of 1.66% and 1.16%, respectively. Thus, the energy absorption capacity substantially increased in the hybrid composites by hybridizing the T and the PE fibers: 59.8 → 175.0 kJ/m^3^ and 58.9 → 109.2 kJ/m^3^, respectively. In addition, the T1.0-SPE1.0 and T1.5-SPE0.5 specimens exhibited the *g*-values of 67.2 and 178.3 kJ/m^3^, respectively, while those of the T1.0-MPE1.0 and T1.5-MPE0.5 specimens were 128.7 and 107.0 kJ/m^3^, respectively. Consequently, it can be noted that the energy absorption capacities of non-hybrid UHPCCs including 2 vol % T, SPE, or MPE fibers were significantly improved by hybridizing the T and SPE or MPE fibers due to their synergic effects.

For the LPE2.0 and LLPE2.0 specimens which showed the relatively high energy absorption capacities, the synergistic effect was not clearly observed. When a portion of the LPE and LLPE fibers was replaced with T fibers, the tensile performance even deteriorated. Consequently, the synergetic effect of the T and PE fibers is considered to be reduced with an increase in the aspect ratio of PE fibers. So, using the longer PE fibers with T fibers is ineffective in terms of energy absorption capacity, might be caused by their higher aspect ratio [3] leading to greater disturbance of fiber dispersion and orientation by existing the T fibers.

#### 3.2.4. Cracking Patterns and Behaviors

**Figure 7:**
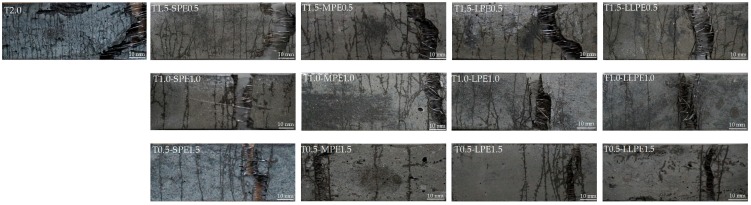
Micro-cracking behaviors of UHPCCs depending on fiber types and volume content.

**Figure 8:**
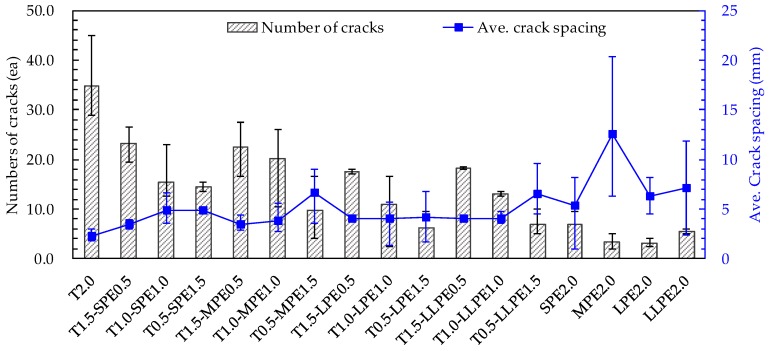
Number of cracks and average crack spacing.

Typical crack patterns of the specimens are shown in Figure 7, while the average cracking behaviors in terms of the number of cracks and average crack spacing of all tested specimens are summarized in Figure 8. The most microcracks were formed in the T2.0 specimens (as many as 35 cracks per sample). The number of cracks decreased as the T fibers were replaced with PE fibers, and this became more significant when the aspect ratio of the PE fibers increased. By replacing 0.5% T fibers with SPE, MPE, LPE, or LLPE fibers, the measured number of cracks was reduced to 23, 23, 18, and 18 per sample, respectively (Figure 7). Subsequently, the number of cracks was further reduced to 16, 20, 11, and 13 per sample by adding 1 vol % PE fibers and 15, 10, 6, and 7 per sample by adding 1.5 vol % PE fibers, respectively. Finally, 7, 4, 3, and 6 cracks per sample were observed with only 2 vol % SPE, MPE, LPE, and LLPE fibers, respectively.

**Figure 9:**
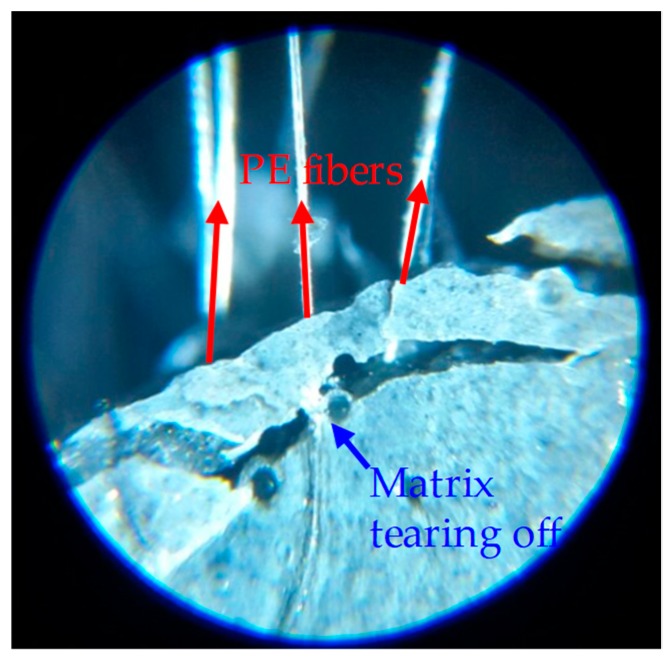
Matrix tearing off phenomenon of PE fibers in UHPCCs.

It is worth noting that the number of microcracks did not show a direct relationship with the tensile strength and especially strain capacity of UHPCCs according to the test results of this study. Although the decrease in tensile strength seemed to correspond with decrease in number of microcracks, its irregularity can be observed by comparing the Figure 4 and Figure 8. For example, the strain capacity of the T2.0 specimen was lowest (0.52%), although the most cracks (35 per sample) were generated. On the contrary, the LLPE2.0 specimen exhibited the high strain capacity of 1.35% but fewer number of cracks (only 6 cracks). The formation of microcracks is related to the pullout mechanisms of the fibers. Since the T fibers are very stiff and have a strong mechanical anchorage effect, they can transfer large level of stress to the surrounding matrix, leading to severe matrix damage and formation of multiple microcracks. Alternatively, the PE fibers were elongated and delaminated by the matrix during pullout due to the lower elastic modulus and surface hardness. The PE fibers are damaged by the matrix rather than damaging the matrix, and this led to the fewer microcracks. The matrix can provide continuous and strong pullout resistance to the PE fibers longer during its pullout. The PE fibers locally tore the matrix off at the localized crack section, as shown in Figure 9. Although the PE fibers cannot crush the cement matrix, their very strong frictional resistance tends to cause local stress concentration and tearing off the matrix. Therefore, it is expected that a proper adjusting the surface condition of the PE fibers would lead to a further improvement in stress distribution and strain capacity of UHPCCs, and additional research needs to be conducted regarding the hybrid effects of this surface treated PE fibers and T fibers on the tensile performance of UHPCCs.

## 4. Conclusions

The hybrid effects of T and PE fibers with four different aspect ratios on the mechanical properties of UHPCCs were investigated. Compressive and tensile tests were conducted for five specimens with single T or PE fibers and thirteen specimens with hybrid T and PE fibers at a constant overall fiber volume fraction of 2%. In addition, the fiber pullout mechanisms at crack surfaces were examined using a microscope to rationally analyze the tensile test results. From the above results and discussions, the following conclusions are drawn:

(1) The compressive strength of UHPCCs increased proportionally to the volume fraction of T fibers. This effect was inversely proportional to the volume fraction of PE fibers, regardless of their aspect ratio.
(2) T fibers were more effective in increasing the tensile strength of UHPCCs than PE fibers, while PE fibers were more effective in improving the strain capacity and energy absorption capacity of UHPCCs.
(3) Using both T and SPE or MPE fibers led to a great synergetic effect that enhanced the tensile strength, strain capacity, and energy absorption capacity of UHPCCs relative to that with only T fibers. However, the specimens with T and LPE or LLPE fibers showed relatively minor improvements in the tensile performance.
(4) For UHPCCs with hybridized T and PE fibers, the tensile performance was deteriorated by increasing the aspect ratio of PE fibers. This effect became more obvious with the higher volume content of PE fibers.
(5) There was no clear relationship between the tensile strain capacity and the number of microcracks. However, the number of microcracks was closely related to the pullout mechanisms of the fibers. The T fibers generated greater number of microcracks than the PE fibers.

## Figures and Tables

**Table 1:** Mixture proportions for UHPCCs.

W/B ^†^	Unit Weight (kg/m^3^)					
	Water	Cement	Silica Fume	Silica Sand	Silica Flour	Superplasticizer *
0.2	160.3	788.5	197.1	867.4	236.6	52.6

* Superplasticizer consists of 30% solid (=15.8 kg/m^3^) and 70% water (=36.8 kg/m^3^). ^†^ W/B was calculated by the ratio of total water content (160.3 kg/m^3^ + 36.8 kg/m^3^) divided by total amount of binder (788.5 kg/m^3^ + 197.1 kg/m^3^).

**Table 2:** Chemical compositions and physical properties of type 1 Portland cement and silica fume.

Composition % (mass)	Cement *	Silica Fume
CaO	61.33	0.38
Al_2_O_3_	6.40	0.25
SiO_2_	21.01	96.00
Fe_2_O_3_	3.12	0.12
MgO	3.02	0.10
SO_3_	2.30	-
Specific surface area (cm^2^/g)	3413	200,000
Density (g/cm^3^)	3.15	2.10

* Type 1 Portland cement.

**Table 3:** Contents of superplasticizer and measured flow values of UHPFRCC mixtures.

Specimen	T2.0	T1.5-SPE0.5	T1.5-MPE0.5	T1.5-LPE0.5	T1.5-LLPE0.5	T1.0-SPE1.0	T1.0-MPE1.0	T1.0-LPE1.0	T1.0-LLPE1.0	T0.5-SPE1.5	T0.5-MPE1.5	T0.5-LPE1.5	T0.5-LLPE1.5	SPE2.0	MPE2.0	LPE2.0	LLPE2.0
Superplasticizer content (g)	470	526	526	526	526	490	526	555	555	670	526	526	526	740	740	740	740
Flow (mm)	170	195	180	180	160	205	175	160	135	120	145	135	130	160	140	145	140

**Table 4:** Physical and mechanical properties of fibers.

Name	*d_f_* (mm)	*L_f_* (mm)	Aspect Ratio (*L_f_*/*d_f_*)	Density (g/cm^3^)	*f_t_* (MPa)	*E_f_* (GPa)
T	0.30	30	100.0	7.9	2428	200
SPE	0.03	12	400.0	0.97	3030	88
MPE	0.03	18	600	0.97	3030	88
LPE	0.03	27	900	0.97	3030	88
LLPE	0.03	36	1200	0.97	3030	88

*d_f_* = fiber diameter, *l_f_* = fiber length, *f_t_* = tensile strength of fiber, *E_f_* = elastic modulus of fiber, T = twisted steel fiber, SPE ~ LLPE = polyethylene fibers with four different aspect ratios.

## References

[1] Kim D.-J., Naaman A.E., El-Tawil S. (2009). High performance fiber reinforced cement composites with innovative slip Hardending twisted steel fibers. Int. J. Concr. Struct. Mater..

[2] Wille K., El-Tawil S., Naaman A.E. (2014). Properties of strain hardening ultra high performance fiber reinforced concrete (UHP-FRC) under direct tensile loading. Cem. Concr. Compos..

[3] Yoo D.Y., Kim S., Park G.J., Park J.J., Kim S.W. (2017). Effects of fiber shape, aspect ratio, and volume fraction on flexural behavior of ultra-high-performance fiber-reinforced cement composites. Compos. Struct..

[4] ACI Committee 239 (2012). Ultra-High Performance Concrete.

[5] Li V.C., Wu C., Wang S., Ogawa A., Saito T. (2002). Interface tailoring for strain-hardening polyvinyl alcohol-engineered cementitious composite (PVA-ECC). ACI Mater. J..

[6] Hassan A.M.T., Jones S.W., Mahmud G.H. (2012). Experimental test methods to determine the uniaxial tensile and compressive behaviour of Ultra High Performance Fibre Reinforced Concrete (UHPFRC). Constr. Build. Mater..

[7] Yoo D.Y., Banthia N., Lee J.Y., Yoon Y.S. (2018). Effect of fiber geometric property on rate dependent flexural behavior of ultra-high-performance cementitious composite. Cem. Concr. Compos..

[8] Wille K., Kim D.J., Naaman A.E. (2011). Strain-hardening UHP-FRC with low fiber contents. Mater. Struct. Constr..

[9] Wu Z., Shi C., He W., Wu L. (2016). Effects of steel fiber content and shape on mechanical properties of ultra high performance concrete. Constr. Build. Mater..

[10] Naaman A.E. (1998). New Fiber Technology. Cem. Ceram. Polym. Compos..

[11] Banthia N., Trottier J.F. (1995). Concrete reinforced with deformed steel fibers. 2. Toughness characterization. ACI Mater. J..

[12] Yao W., Li J., Wu K. (2003). Mechanical properties of hybrid fiber-reinforced concrete at low fiber volume fraction. Cem. Concr. Res..

[13] Park J.J., Kang S.T., Koh K.T. Influence of the ingredients on the compressive strength of UHPC as a fundamental study to optimize the mixing proportion. Proceedings of the Second International Symposium on Ultra High Performance Concrete.

[14] Ma J., Orgass M., Dehn F., Schmidt D., Tue N.V. Comparative Investigations on Ultra-High Performance Concrete with or without Coarse Aggregates. Proceedings of the International Symposium on Ultra High Performance Concrete.

[15] Orgass M., Klug Y. Fibre Reinforced Ultra-High Strength Concretes. Proceedings of the International Symposium on Ultra High Performance Concrete.

[16] Collepardi S., Coppola L., Troli R., Collepardi M. (1997). Mechanical properties of modified reactive powder concrete. ACI Spec. Publ..

[17] Graybeal B., Davis M. (2008). Cylinder or Cube Strength Testing of 80 to 200 MPa Ultra-High-Performance Fiber-Reinforced-Concrete. ACI Mater. J..

[18] (2013). ASTM C1437. Standard Test Method for Flow of Hydraulic Cement Mortar.

[19] (1998). ASTM C 39-96, Standard test method for compressive strength of cylindrical concrete specimens. Am. Soc. Test. Mater..

[20] Japan Society of Civil Engineers (JSCE) (2004). Recommendations for Design and Construction of Ultra High Performance Fiber Reinforced Concrete Structures (Draft).

[21] Rizzuti L., Bencardino F. (2014). Effects of fibre volume fraction on the compressive and flexural experimental behaviour of SFRC. Contemp. Eng. Sci..

[22] Kim M.J., Yoo D.Y., Kim S., Shin M., Banthia N. (2018). Effects of fiber geometry and cryogenic condition on mechanical properties of ultra-high-performance fiber-reinforced concrete. Cem. Concr. Res..

[23] Yoo D.Y., Shin H.O., Yang J.M., Yoon Y.S. (2014). Material and bond properties of ultra high performance fiber reinforced concrete with micro steel fibers. Compos. Part B Eng..

[24] Atiş C.D., Karahan O. (2009). Properties of steel fiber reinforced fly ash concrete. Constr. Build. Mater..

[25] Li V.C., Leung C.K.Y. (1992). Steady-State and Multiple Cracking of Short Random Fiber Composites. J. Eng. Mech..

[26] Naaman A.E. Toughness, ductility, surface energy and deflection-hardening FRC composites. Proceedings of the JCI International Workshop on Ductile Fiber Reinforced Cementitious Composites—Application and Evaluation.

[27] Martinie L., Roussel N. (2011). Simple tools for fiber orientation prediction in industrial practice. Cem. Concr. Res..

